# Generation of induced pluripotent stem cell-derived beta-cells in blood amino acids-like medium

**DOI:** 10.1242/bio.059581

**Published:** 2023-03-21

**Authors:** Marwa Ali, Yusuke Kato, Nobuaki Shiraki, Shoen Kume

**Affiliations:** School of Life Science and Technology, Tokyo Institute of Technology, 4259-B-25 Nagatsuta-cho, Midori-ku, Yokohama, Kanagawa 226-8501, Japan

**Keywords:** Human induced pluripotent stem cell (hiPSCs), Pancreatic endocrine beta-cells, Amino acids

## Abstract

Traditional cell culture media do not accurately represent the availability of the nutrients in plasma. They usually contain a supraphysiological concentration of nutrients such as glucose, amino acids, etc. These high nutrients can alter the metabolism of cultured cells and induce metabolic phenotypes that do not reflect *in vivo* conditions. We demonstrate that the supraphysiological levels of nutrients interfere with endodermal differentiation. Refinement of media formulations has a potential application in maturity modulation of stem cell-derived β-cells (SC-β) generation *in vitro*. To address these issues, we established a defined culture system to derive SC-β-cells using a blood amino acids-like medium (BALM). Human induced pluripotent stem cells (hiPSCs) can be efficiently differentiated into the definitive endoderm, pancreatic progenitors, endocrine progenitors, and SC-β in BALM-based med. The differentiated cells secreted C-peptide *in vitro* in response to high glucose levels and expressed several pancreatic β-cell markers. In conclusion, amino acids at the physiological levels are sufficient for deriving functional SC-β cells.

## Teaser

**Summary:** We used a blood amino acids-like medium and found that it is useful to efficiently differentiate human iPSCs into the definitive endoderm, and to generate functional pancreatic beta-cells.

## INTRODUCTION

Previous reports have shown that deprivation of nutrients can eliminate residual undifferentiated cells and potentiate differentiation. This potential of metabolic approaches for modulating differentiation efficiency and the initial cell fate specification receives attention (Dahan et al., 2019; Shiraki et al., 2014; Tohyama et al., 2013, 2016; Wu et al., 2016).

While metabolism offers a potential approach to improving the efficacy of cellular therapy, traditional cell culture media do not accurately represent the nutrients available in plasma. They usually contain supraphysiological concentrations of nutrients such as glucose, amino acids, etc. (Yao and Asayama, 2017). These high nutrients can alter the metabolism of cultured cells and induce metabolic phenotypes that do not reflect *in vivo* conditions (Ackermann and Tardito, 2019). Efforts in cancer research to create culture media that resemble nutrient and metabolite levels found in human plasma have shown differences in metabolic phenotype and drug efficacy compared to conventional media (Cantor et al., 2017; Leney-Greene et al., 2020; Rossiter et al., 2021; Vande Voorde et al., 2019). Refinement of media formulations can potentially improve the efficiency of the *in vitro* generation of pancreatic β-cells from human induced pluripotent stem cells (hiPSCs).

Several recent protocol modifications to improve the maturation and homogeneity of hiPSCs-derived β (SC-β) cells were achieved by altering culture conditions for differentiation or using small-molecule screening (Velazco-Cruz et al., 2019; Velazco-Cruz et al., 2020; Veres et al., 2019). Metabolic shifts in β-cells were previously reported to correlate with maturation (Davis et al., 2020; Wortham et al., 2018). These suggest that β-cell maturation may not only be transcriptionally determined but also subjected to nutritional and environmental changes (Helman et al., 2020; Vanderkruk and Hoffman, 2021; Zeng et al., 2017). Previous reports from mice studies showed that amino acid levels progressively decreased between embryonic day 19 and postnatal day 9. It is demonstrated that late-stage culture from day 20 for 14-20 days in low levels of amino acids promoted the maturation of SC-β cells and improved their glucose-stimulated insulin secretion (GSIS) function (Helman et al., 2020). However, the effect of the amino acids at physiological levels on the whole differentiation process has not been investigated. In this study, we asked whether SC-β cells could be derived in media that better recapitulates the composition of human plasma amino acids and whether it would promote differentiation.

To address these issues, we used a culture system to derive SC-β cells using a blood amino acids-like medium (BALM). We demonstrated that supraphysiological nutrient levels interfere with endodermal differentiation. hiPSCs can be efficiently differentiated into DE, pancreatic progenitors, endocrine progenitors, and SC-β in the BALM-based medium. The differentiated cells secreted C-peptide *in vitro* in response to various insulin secretagogues and high glucose levels and expressed several pancreatic β-cell markers.

## RESULTS

### hiPSCs are efficiently differentiated into definitive endoderm (DE) in the BALM-based medium

**Fig. 1.**
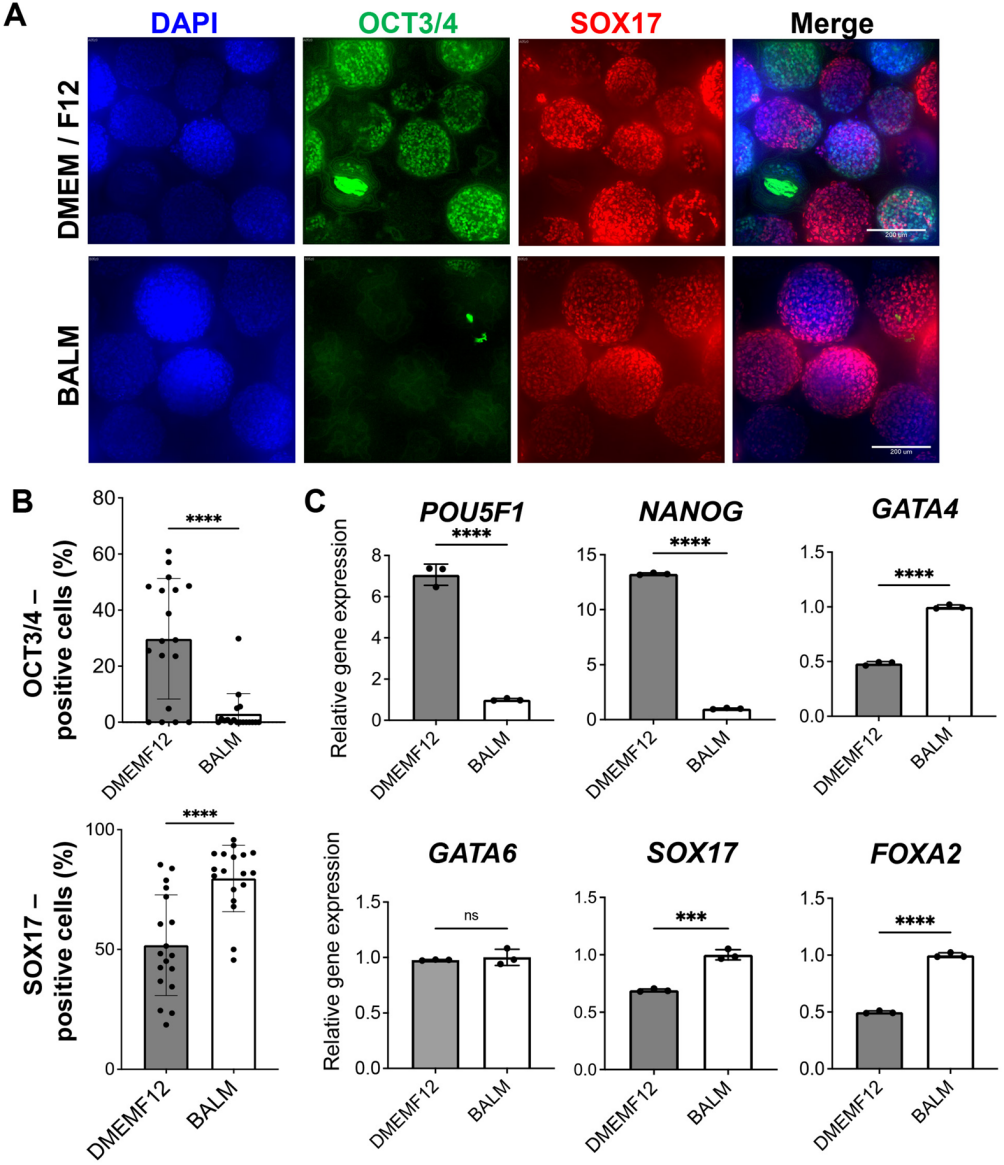
**BALM improved definitive endoderm (DE) differentiation efficiency compared to conventional DMEM F12 media *In vitro* differentiation of hiPSCs cells into DE cells in BALM medium.** (A) Immunocytochemical analysis of hiPSCs-derived cell clusters cultured in DMEM F12 (upper) or BALM (lower) based media on differentiation day 3, stained with DE marker SOX17 (red), pluripotent marker OCT3/4 (green), and nuclei (DAPI, blue). Representative images are shown. Scale bars: 200 µm. (B) Quantification of OCT3/4 (upper) and SOX17 (lower)-positive day-3 cell clusters cultured in DMEM F12 or BALM-based medium. Y-axis, the % positivity of immunostainings. Each dot represents one cluster. (C) Expression of marker genes for pluripotency (*NANOG*, *POU5F1*) and DE (*GATA4*, *GATA6*, *SOX17*, *FOXA2*) in iPSC-derived day-3 clusters cultured in DMEM F12 or BALM, analyzed by real-time PCR analyses. Y-axis, fold changes compared to those cultured in BALM (=1). Data are expressed as mean±s.d. *N*=3 biological replicates. Differences between groups were analyzed by Student's *t*-test; significances are shown as *P<0.05, **P<0.01, ***P<0.001 or ****P<0.0001.

The concentrations of amino acids in the media used for cell cultures, such as DMEM F12, are much higher than those found in the blood and therefore do not seem to reflect *in vivo* conditions. We refer to a report to develop a chemically defined medium with amino acids and glucose at concentrations generally found in human plasma (Matsumoto et al., 2014) and tested a custom-made blood amino acids-like medium (BALM). The comparison of the formulation of BALM and DMEM F12 with the blood amino acid is shown in Table S1**.** We compared using BALM and DMEM F12-based medium and assayed for their effects on differentiation of the human-induced pluripotent stem cells (hiPSCs) RPChiPS771 cells. To examine the impact of BALM on differentiation, we assessed SOX17 and OCT3/4 expression of day 3 clusters. Immunofluorescence revealed an increase in the ratio of SOX17-positive cells cultured in BALM compared to those cultured in DMEM F12-based medium. OCT3/4-positive cells significantly reduced when hiPSCs were differentiated in BALM compared to DMEM F12 (Fig. 1A,B). Expression analysis of the pluripotency markers (*POU5F1*, *NANOG*) showed significantly decreased levels in cells cultured in BALM compared to those cultured in DMEM F12-based medium. The definitive endoderm (DE) marker genes (*GATA4*, *SOX17*, *FOXA2*) revealed a significant increase in cells cultured in BALM compared to those in DMEM F12 (Fig. 1C). The BALM-based endoderm differentiation protocol was beneficial in that potentiation of differentiation could be achieved compared to the DMEM F12-based conventional medium.

Despite BALM having been designed and developed independently, a human plasma-like medium (HPLM) (Cantor et al., 2017) was reported during our study. The composition of HPLM is also shown in Table S1. We then tested HPLM and compared it with a BALM-based medium in the endodermal differentiation of hiPSCs (Fig. S1). *POU5F1* and *NANOG* expressions were reduced in iPSC-derived endodermal cells cultured in HPLM or BALM compared to those in DMEM F12. In contrast, expressions of DE marker genes, *GATA4*, *GATA6*, *SOX17,* and *FOXA2*, increased significantly in cells differentiated in BALM or HPLM compared to DMEM F12 (Fig. S1). The result thus cross-validates our hypothesis of physiological nutrients’ role in differentiation enhancement.

### hiPSCs differentiated in the BALM-based medium can give rise to pancreatic progenitor cells, endocrine progenitor cells, and endocrine cells

**Fig. 2.**
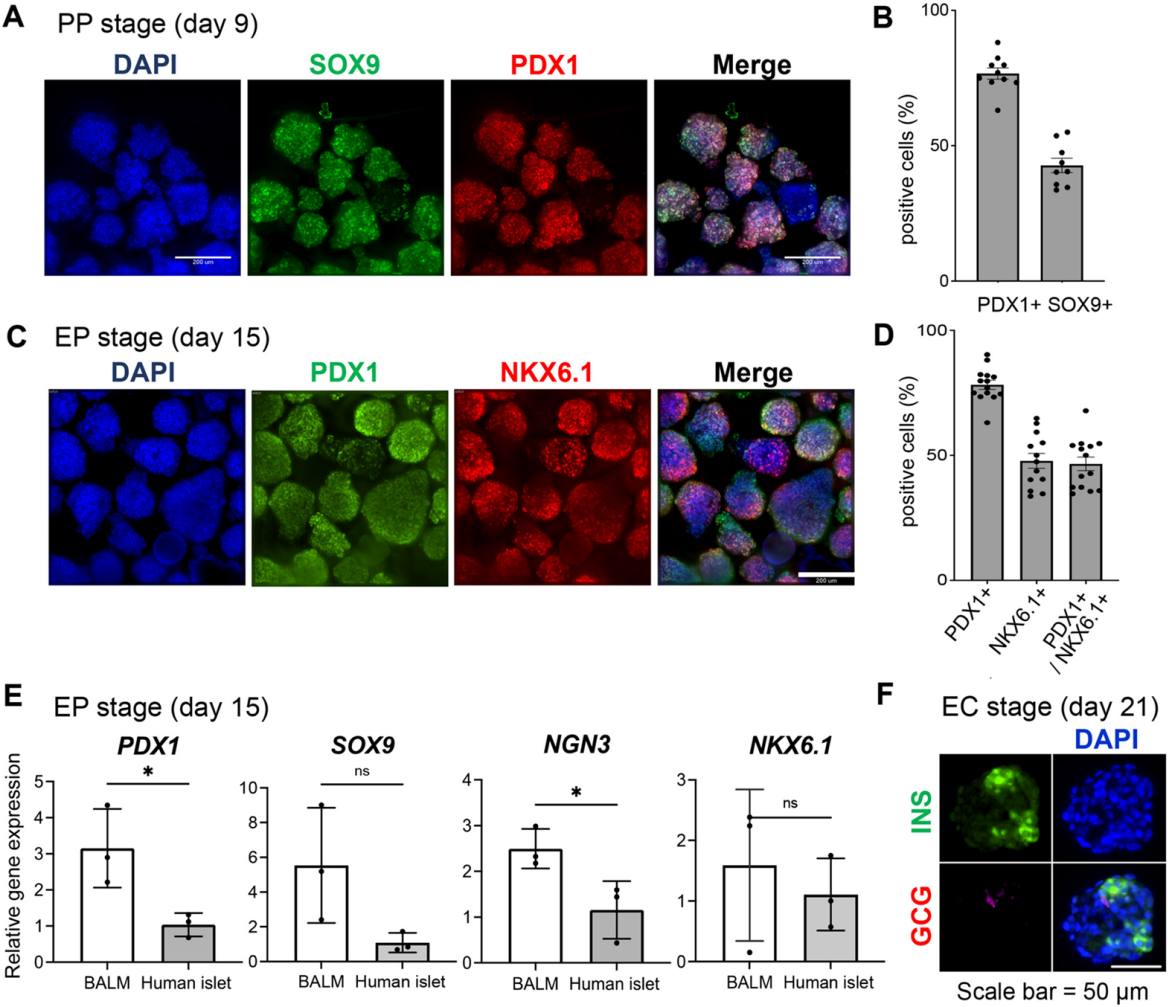
**Differentiation in BALM-based medium can give rise to pancreatic progenitor cells, endocrine progenitor cells and endocrine cells.** (A,B) Immunocytochemical analysis of stage-3 hiPSC-derived PP cells generated with BALM-based medium on day 9. Cell clusters were stained for PP marker PDX1 (red), SOX9 (green), and nuclei (DAPI, blue). (C,D) Immunocytochemical analysis of stage-4 EP on day 15. Cell clusters were stained for EP marker NKX6.1 (red), PDX1 (green), and nuclei (DAPI, blue). (A,C) Representative images are shown. Scale bars: 200 µm. (B,D) Each dot represents one cluster. (E) Real-time PCR analyses of pancreatic differentiation marker genes on differentiation day 15. Y-axis, fold changes compared to human islets (=1). (F) Immunocytochemical analysis of stage 5 EC on day 21. Cell clusters were stained for EC markers INS (green), GCG (red), and nuclei (DAPI, blue). Scale bar: 50 µm. Data are expressed as mean±s.d. *N*=3 biological replicates. Differences between groups were analyzed by Student's *t*-test; significances are shown as *P<0.05, **P<0.01, ***P<0.001 or ****P<0.0001.

Next, we determined whether the culture in a BALM-based medium permits differentiation into PDX1+ pancreatic progenitor (PP). hiPSC-derived DE cells were differentiated into PP cells (day 9). Differentiation of hiPSC in BALM-based medium yielded (76.0%±7.6%) PDX1+ PP cells and (42.7%±7.4%) SOX9+ cells (Fig. 2A,B). Further differentiation of PP cells into endocrine progenitor (EP) cells yielded approximately (46.0%±10.4%) PDX1/ NKX6.1 double-positive cells on day 15 (Fig. 2C,D). hiPSC-derived EP cells (day 15) expressed pancreatic progenitor marker genes, *PDX1*, *SOX9*, and endocrine progenitor marker genes, *NGN3* and *NKX6.1* (Fig. 2E). The higher expression levels of these genes in the hiPSC-derived EP cells than those found in human islets suggesting their immature nature. On day 21, INS+ endocrine cells (EC) gave rise, and the majority were INS single-positive cells (Fig. 2F). The results show that hiPSC-derived DE can be efficiently differentiated into the PP, EP and EC cells under the BALM-based medium.

### BALM-based medium enables the derivation of glucose-responsive SC-β Cells

**Fig. 3.**
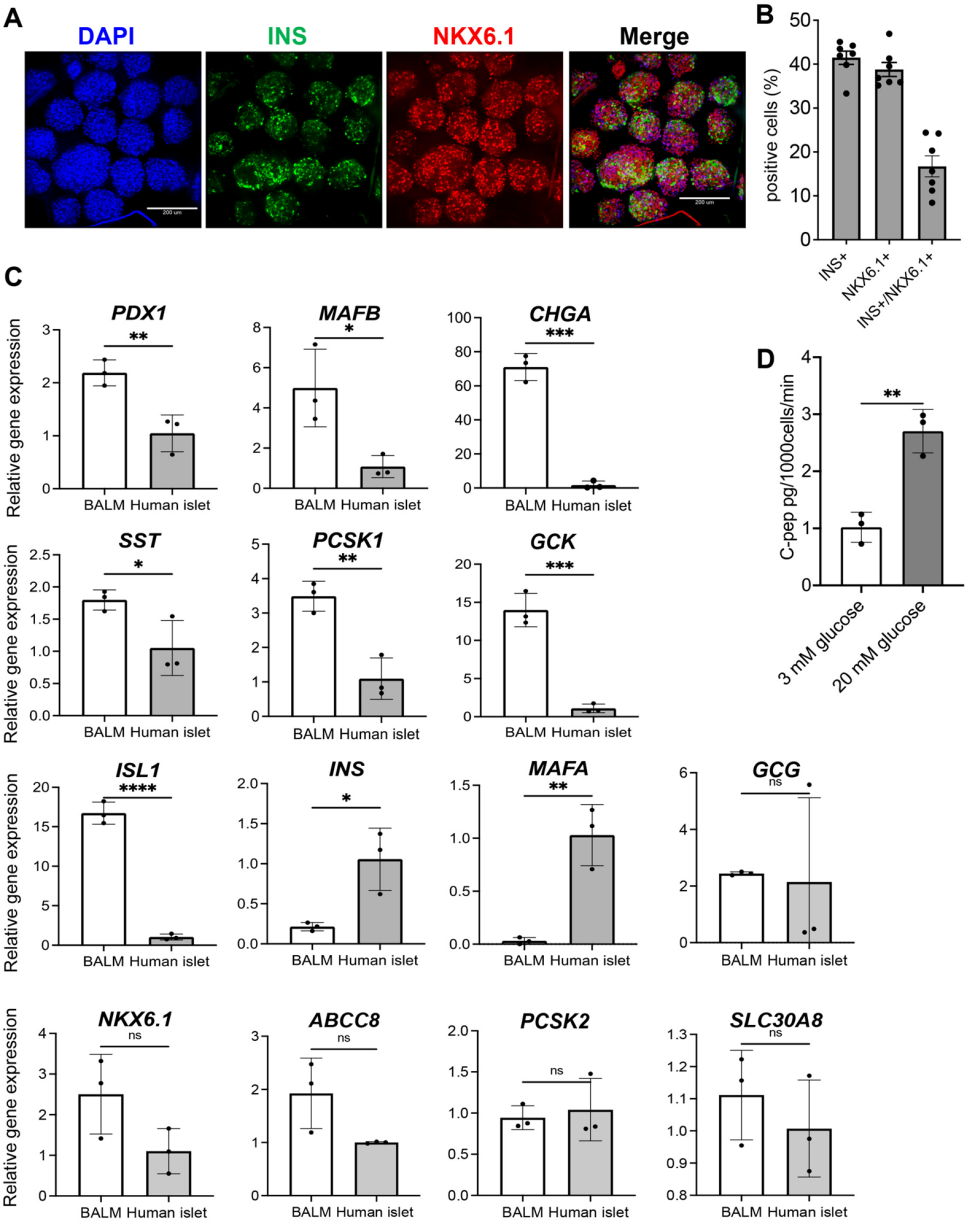
**BALM-based medium enables derivation of SC-β Cells.** (A,B) Immunocytochemical analysis of stage 6 SC-β generated with BALM-based medium on day 33. Cell clusters were stained for SC-β marker NKX6.1 (red), INS (green), and nuclei (DAPI, blue). Representative images are shown. Scale bar: 200 µm. (B) Y-axis, the % positivity of staining. Each dot represents one cluster. (C) Real-time PCR analyses of pancreatic endocrine differentiation marker genes on day 33. Y-axis, fold changes compared to human islets (=1). (D) Glucose-stimulated insulin secretion of SC-β cells differentiated by culturing in BALM-based medium. Data are expressed as mean±s.d. *N*=3 biological replicates. Differences between groups were analyzed by Student's *t*-test; significances are shown as *P<0.05, **P<0.01, ***P<0.001 or ****P<0.0001.

We next examined the differentiation into insulin-expressing SC-β cells. Immunostaining of SC-β cells on differentiation day 33 revealed (40.7%±4.9%) INS+ cells, (39.2%±4.3%) NKX6.1+ cells, and (18.9%±5.0%) INS/NKX6.1 double-positive cells (Fig. 3A,B). We examined the expression of several β cells and islet markers in SC-β cells and compared them with cadaveric human islets. Our SC-β cells exhibited higher *PDX1*, *MAFB*, *CHGA*, *SST*, *PCSK1*, *GCK*, and *ISL1* levels than human islets. *INS* and *MAFA* expressions were lower than those of the human islets. *GCG*, *NKX6.1*, *ABCC8*, *PCSK2,* and *SLC30A8* expressions were comparable with human islets. Gene expressions are presented as fold-changes compared to human islets (human islet=1) (Fig. 3C). The results are consistent with previously published results of stage 6 cells where the expressions of several β cell markers were equal to or greater than human islets. However, other markers remain low (Velazco-Cruz et al., 2019).

We tested the function of stage 6 cells generated in a BALM-based medium using a static GSIS assay. The SC-β cells secreted insulin when challenged with high glucose (Fig. 3D). The data suggest that our BALM-based differentiation is suitable for pancreatic beta-cell differentiation.

## DISCUSSION

The presence of undifferentiated cells that might interfere with the function of the desired cell types or even be carcinogenic remains a challenging issue with hiPSCs-derived therapeutic products. Improving the efficiency of the generation of glucose-responsive cell populations while minimizing undesired cell types during *in vitro* differentiation is crucial for the clinical translation of SC-β cells toward curative diabetes treatment (Nair et al., 2020). Exploiting metabolic dependence by eliminating or restricting nutrient availability is emerging as a novel cancer therapeutic module (Nair et al., 2020; Pavlova and Thompson, 2016; Wang et al., 2019).

Many of the conventional cell-culture media are established to sustain continuous cancer cell proliferation *in vitro*. Yet, their formulation does not reflect the nutritional environment of our body fluid. Recent reports show that culturing cells in media with supraphysiological concentrations of nutrients leads to recapitulating more cancer-cell-like metabolism. In cancer research, efforts have been undertaken to develop culture media that mimic the amounts of nutrients and metabolites in human plasma. Compared to traditional media, such investigations have revealed changes in metabolic phenotype and therapeutic efficacy (Cantor et al., 2017; Leney-Greene et al., 2020; Rossiter et al., 2021; Vande Voorde et al., 2019). Refinement of media formulations can potentially improve the efficiency of the *in vitro* generation of pancreatic β-cells from hiPSCs. The metabolic signature of hiPSCs is highly similar to cancer cells. Both depend on aerobic glycolysis, increased amino acid uptake, and glutamine oxidation as sources of energy expenditure and biomass production (Intlekofer and Finley, 2019).

This study shows that through refinements of the nutrient composition of BALM-based medium, we successfully promoted hiPSCs differentiation into the DE. The physiological-like culture medium formulation significantly reduced the undifferentiated cell population. Moreover, pluripotency markers (*POU5F1*, *NANOG*) expression was considerably lower in cells cultured in BALM than in those cultivated in DMEM F12-based medium. When comparing cells grown in BALM to those cultured in DMEM F12, the DE marker genes (*SOX17*, *FOXA2*, *GATA4*) exhibited a significant increase in cells cultured in a BALM-based medium. Our findings indicate that supraphysiological concentrations of nutrients suppressed DE differentiation. The use of physiological levels of nutrients can lead to modulating cell function.

Our finding may have potential translational application in developing hPSCs models for studying metabolic regulation of pancreatic β-cell differentiation (Balboa et al., 2019; Tyurin-Kuzmin et al., 2020). In conclusion, we developed a specified culture system to generate SC-β cells using a blood amino acids-like medium (BALM). BALM-based medium successfully converts hiPSCs into the definitive endoderm, pancreatic progenitors, endocrine progenitors, and SC-β. It would be interesting to study the future long-term metabolic changes of the hiPSCs.

## MATERIALS AND METHODS

### Ethics approval

The use of hiPSCs was approved by the institutional ethical committee involving human materials by the Tokyo Institute of Technology.

### Maintenance culture of human iPS cells

RPChiPS771 human induced pluripotent stem cells (iPSCs; REPROCELL) were maintained in StemFit AK02N medium (Ajinomoto) on 100 mm CellBIND cell culture dishes (Corning, 3296) precoated with vitronectin (ThermoFisher Scientific, A31804). Cell passages were performed as follows. hiPSCs were washed, dissociated with TrypLE^TM^ Select (Gibco), and resuspended in StemFit AK02N medium supplemented with 10 µM ROCK inhibitor (Y-27632; Wako) and replated at 1-2×10^6^ cells per dish.

### Endodermal differentiation of hiPSCs

For spheroid formation, RPChiPS771 human iPSCs were harvested, dissociated, and replated at a density of 5×10^6^ cells per well onto six-well suspension culture plates (Greiner Bio One, 657185) in AK02N medium supplemented with 10 µM ROCK inhibitor, and cultured at 37°C for 24 h to form sphere on a rotating orbital shaker at 95 RPMs. Endodermal differentiation was initialized 24 h post-seeding by changing with differentiation medium (Fig. 1). Custom-made BALM, DMEM F12 and HPLM were used for differentiation (Institute of peptide research) (Fig. S1).

### Pancreatic differentiation of hiPSCs

We performed pancreatic differentiation following a previously reported protocol with modifications (Velazco-Cruz et al., 2019). RPChiPS771 human iPSCs were harvested, dissociated, and replated at a density of 5×10^6^ cells per well onto six-well suspension culture plates in BALM-based medium supplemented with differentiation factors as follows. Stage 1 (3 days): S1 medium + 100 ng/ml Activin A (cell guidance system GFH6-1000) + 3 μM Chir99021 (Wako 034-23103) + 10 µM Y27632 for 1 day. S1 medium + 100 ng/ml Activin A for 2 days. Stage 2 (3 days): S2 medium + 50 ng/ml KGF (KGF Wako, 116-00811). Stage 3 (2 day): S3 medium + 50 ng/ml KGF + 200 nM LDN193189 (WAKO SML0559-5MG) + 500 nM PdBU (LC Laboratories, P-4833) + 2 μM Retinoic Acid (Stemgent 04-0021) + 0.25 μM Sant1 (Sigma, S4572) + 10 µM Y27632. Stage 4 (5-6 days): S4 medium + 5 ng/ml Activin A + 50 ng/ml KGF + 0.1 µM Retinoic Acid + 0.25 µM SANT1 + 10 µM Y27632. Stage 5 (8 days): S5-1 medium (4 days) + 10 µM ALK5i II (TOCRIS 3742) + 20 ng/ml Betacellulin (PrepoTech, 100-50) + 0.1 µM Retinoic Acid + 1 µM T3 (Sigma, T6397) + 1 µM XXI (Sigma 595790), 10 µM Heparin + 0.25 µM SANT1 + 10 µM Y27632. S5-2 (4 days) medium + 10 µM ALK5i II (TOCRIS 3742) + 20 ng/ml Betacellulin (PrepoTech, 100-50) + 0.1 µM Retinoic Acid + 1 µM T3 (Sigma, T6397) + 1 µM XXI (Sigma, 595790) 10 µM Heparin + 10 µM Y27632. Stage 6 (14 days): on the first day of stage 6, reaggregation of cell spheres was performed. Cell spheres were washed in PBS and incubated in TrypLE Select Enzyme (ThermoFisher Scientific, 12563029) for 10 min at 37C. Spheres were mechanically dissociated using a P1000 pipette. Cells were washed with S6 medium + Y27632 (10 µM), resuspended in S6 medium + Y27632, then passed through a 100-µm CellStrainer (pluriSelect) to remove any residual undissociated clusters. The dissociated single cells were counted and seeded into a six-well suspension culture plate (Greiner Bio One, 657185). Differentiation media formulations used were the following. S1 medium: BALM supplemented with 1% Penicillin-Streptomycin solution (PS; Nacalai Tesque), 1% (v/v) B27 insulin (-) (Life Technologies, A1895601), Recombinant IGF1 100 ng/ml (Oriental Yeast). S2 medium: BALM supplemented with 1% PS, 1% (v/v) B27 (Life Technologies, 17504044), S3 medium: BALM supplemented with 1% PS, 1% (v/v) B27 (Life Technologies, 17504044), S4 medium: BALM supplemented with 1% PS, 1% (v/v) B27 minus vitamin A (Life Technologies, 12587010), S5 medium: BALM supplemented with 1% PS, 1% (v/v) (B27 minus vitamin A, 3.6 g/L glucose (Otsuka Pharmaceutical Factory). S6 medium: BALM supplemented with 1% PS, 2% (w/v) fatty acid-free (FAF)-BSA (Proliant Biologicals, 68700-100G), 0.44 g/L glucose, 1 µM ZnSO4 (Sigma, Z0251-100G), 10 µM Heparin, 0.1% Trace Elements A (Corning, 25-021-CI), and 0.1% Trace Elements B (Corning, 25-022-CI).

### Real-time PCR analysis

RNA was extracted from human iPS cells using the RNeasy mini-kit, or All prep (DNA/RNA) Mini Kit (Qiagen, Germany) and then treated with DNaseI (Qiagen). 200 ng RNA was reverse-transcribed using PrimeScript™ RT Master Mix (Takara, Japan). For real-time PCR analysis, mRNA expressions were quantified with SyberGreen on a StepOne Plus (Applied Biosystems, Foster City, CA, USA).

The PCR conditions were as follows: initial denaturation at 95°C for 30 s, denaturation at 95°C for 5 s, annealing and extension at 60°C for 30 s, for up to 40 cycles. Target mRNA levels were expressed as arbitrary units and were determined using the ΔΔ CT method.

Primer sequences used for the detection of endoderm and pancreatic differentiation and internal controls are listed in Table S2.

For human islet cDNA three batches of first strand cDNA from non-diabetic human islets (Cosmo Bio): HIcDNA149, HIcDNA171T, and HIcDNA171) were used.

### Immunocytochemistry

hiPSC-derived cell clusters were fixed with 4% paraformaldehyde (Nacalai Tesque) in phosphate-buffered saline (PBS), permeabilized with 1% Triton X-100, and blocked with 20% Blocking One (Nacalai Tesque 03953-66) and PBS-T (0.1% Tween-20 in PBS) for 1-2 h. After washing the cells in PBS-T three times, cells were counterstained with 4′, 6-diamidino-2-phenylindole (DAPI) (Roche Diagnostics). Images were captured using an ImageXpress Micro scanning system (Molecular Devices, Japan). Quantitative analysis of positive cells versus total cells (DAPI-positive cells) was performed using the MetaXpress cellular image analysis software (Molecular Devices). The primary and secondary antibodies used are summarized in Table S3.

### Glucose-stimulated insulin secretion (GSIS) assay

C-peptide release and C-peptide content assay were performed as described previously with minor modifications (Velazco-Cruz et al., 2019). Briefly, differentiated cells were loaded on Transwell (Corning, 3415). Plates were allowed to fast for 60 min in 3 mM glucose HKRB (HEPES Krebs-Ringer Bicarbonate buffer) with 0.2% BSA, followed by another wash step and replacement of KRB with low, high glucose at 37°C on an orbital shaker (95 rpm). The collected culture supernatant was sampled and stored at −80°C until analysis. Cells were lysed, and RNA and DNA were purified using AllPrep DNA/RNA Micro Kit (Qiagen). C-peptide secretion was measured using a human C-peptide ELISA Kit (Mercodia) and a human C-peptide AlphaLISA kit (PerkinElmer). The amount of C-peptide was normalized to total DNA contents in the corresponding cell lysate. DNA contents corresponding to 1000 cells were used for normalization. Fresh HKRB was prepared for each experiment.

### Quantification and statistical analysis

Individual data are shown or expressed as the mean± standard deviation (s.d.). Each experiment was conducted with three biological replicates (*N*=3) and repeated multiple times. Differences between groups were analyzed by Student's *t*-tests; differences are shown as *P<0.05, **P<0.01, ***P<0.001 or ****P<0.0001.

## Supplementary Material

10.1242/biolopen.059581_sup1 Supplementary information
