# Supplementary material for: Generation of induced pluripotent stem cell-derived beta-cells in blood amino acids-like medium
Source: Biol Open. 2023 Mar 21;12(3):bio059581. doi: 10.1242/bio.059581 (PMC10084857; doi:10.1242/bio.059581)
Supplement: Supplementary information [file biolopen-12-059581-s1.pdf]

Fig. S1

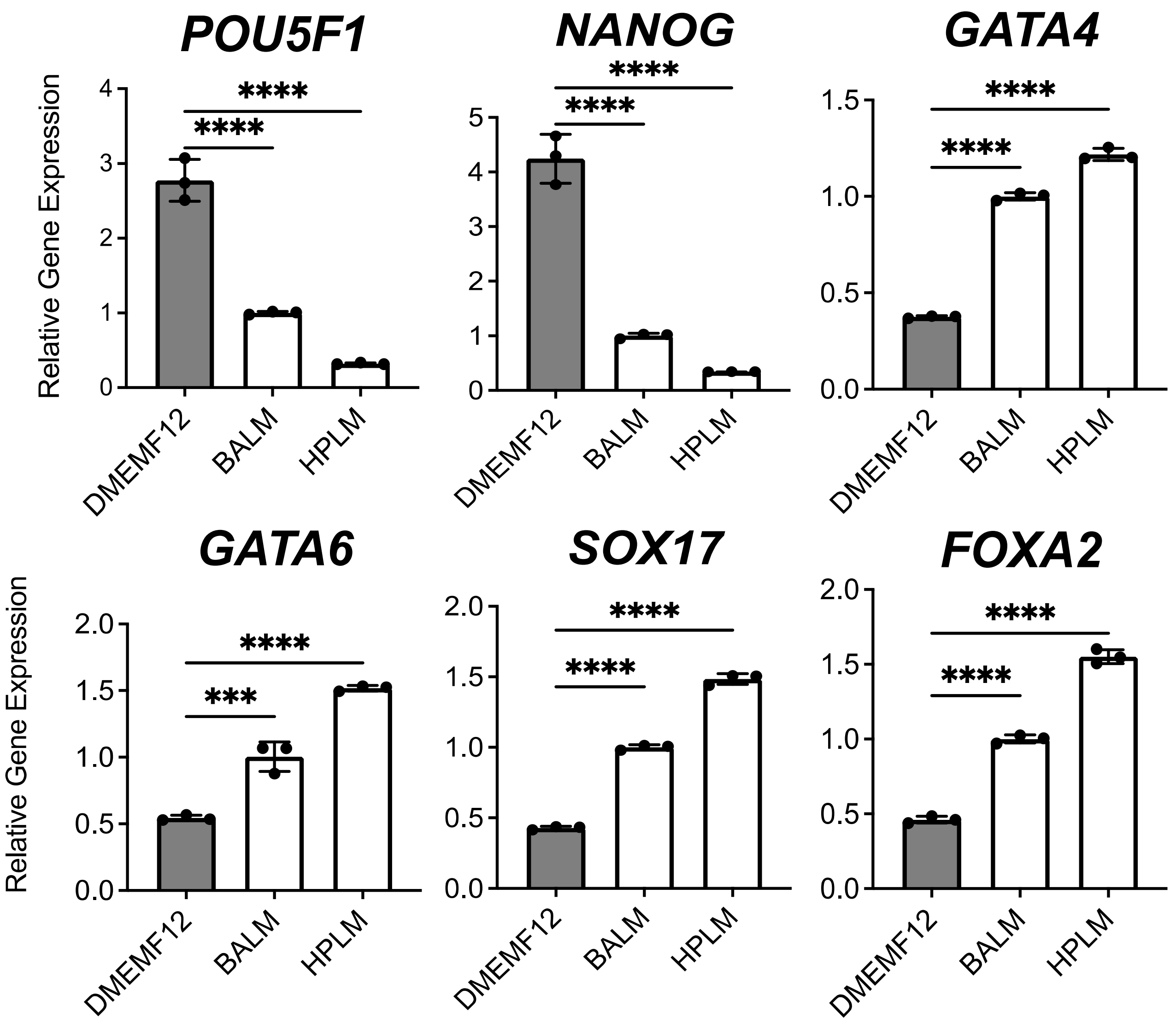

Fig. S1. *In vitro* differentiation of hiPS cells into DE, cells in BALM and HPLM medium RT-PCR expression of marker genes for DE (*GATA4*, *GATA6*, SOX17*, FOXA2*) and pluripotency (*NANOG*, *POU5F1*)

Biology Open • Supplementary information

Table S1. Continued

Other Components [μM]	DMEM F12	BALM	HPLM
D-Glucose	16666.8	5555.6	5000
Hypoxanthine	15.0331	15.0331	10
Linoleic Acid	0.1497593	0.1497593	NA
Lipoic Acid	0.5089675	0.5089675	NA
Phenol Red	21.5197	21.5197	14
Putrescine 2HCl	0.5028869	0.5028869	NA
Na Pyruvate	500	500	50
Thymidine	1.6515	1.6515	NA
2-hydroxybutyrate	NA	NA	50
3-hydroxybutyrate	NA	NA	50
4-hydroxyproline	NA	NA	20
Acetate	NA	NA	40
Acetone	NA	NA	60
Acetylglycine	NA	NA	90
Alpha-aminobutyrate	NA	NA	20
Betaine	NA	NA	70
Carnitine	NA	NA	40
Citrate	NA	NA	130
Citrulline	NA	NA	40
Creatine	NA	NA	40
Creatinine	NA	NA	75
Formate	NA	NA	50
Fructose	NA	NA	40
Galactose	NA	NA	60
Glutathione	NA	NA	25
Glycerol	NA	NA	120
Hypoxanthine	NA	NA	10
Lactate	NA	NA	1600
Malonate	NA	NA	10
Ornithine	NA	NA	70
Succinate	NA	NA	20
Taurine	NA	NA	90
Urea	NA	NA	5000
Uric acid	NA	NA	350

The compositions of DMEM F12 and BALM are shown. For human plasma, only the amino acid concentrations are shown (Matsumoto et al, 2014).

Biology Open • Supplementary information

Table S2. Primer sequences used for real-time PCR analyses.

Gene	Primer Sequence (Forward)	Primer Sequence (Reverse)
ACTB	TGGCACCCAGCACAATGAA	CTAAGTCATAGTCCGCCTAGAAGCA
FOXA2	CGTCCGACTGGAGCAGCTACTAT	ATGTACGTGTTCATGCCGTTCA
GATA4	TGGTCAGATGGCAGCCAGAG	TGCTTCGAATTCGTGTTGCAG
GATA6	GCAATAATTCCATTCCCATGAC	AGCCCATCTTGACCCGAATA
GAPDH	GCACCGTCAAGGCTGAGAAC	TGGTGAAGACGCCAGTGGA
NANOG	TCCAACATCCTGAACCTCAGCTA	TGCGTCACACCATTGCTATTCTT
POU5F1	TGAAGCTGGAGAAGGAGAAGCTG	GCAGATGGTCGTTTGGCTGA
SOX17	CTGCAGGCCAGAAGCAGTGTTA	CCCAAACTGTTCAAGTGGCAGA
INS	GAAGCGGCATTGTGGAAC	CTGGTTCAAGGGCTTTATTCCATC
ISL1	ACAAAGTTACCAGCCACCTTGGA	TCATGCCTCAATAGGACTGGCTAC
PCSK1	CTCACCTCACCTGGATGATCACTAA	CCAGCATTGTAGGTGACTGGAGAC
NGN3	GCGAGTTGGCACTGAGCAAG	CCGAGTTGAGGTTGTGCATTC
PCSK2	CAATGCACATCCATTCCCAAG	AAGGTGCTCCAGGCTAAGCTC
SST	CCCAGACTCCGTCAGTTTC	GGACAGATCTTCAGGTTGGAG
NKX6.1	GAGGGCTCGTTTGGCCTATTC	ATCTCGGCAGCGTGCTTCT
SOX9	GGAGATGAAATCTGTTCTGGGAATG	TTGAAGGTTAACTGCTGGTGTTCTG
CHGA	TCCCTGTGAACAGCCCTATGAATAA	AAAGTGTGTCGGAGATGACCTCAA
MAFB	TTGTAACCAGAATCACCCTGAGGTC	CCAGGGTCAGGGATGGCTAA
GCK	ATGACCGGCACTGCTGAGA	AGCCCTTGGTCCAGTTGAGAA
GCG	CTTGCCGCCAGGGACTTTA	ACGTGGCTAGCAGGTGATGTTG
ABCC8	TCACACTTTGCCGAAACCGTAG	ATCTGTTGGCAGCTGTGAGGA
PDX1	ACTCCACCTTGGGACCTGTTTAGA	CGAGTAAGAATGGCTTTATGGCAGA
MAFA	TTGAGCCAGGTCTAACTTCTTTCCA	AAGGTGGGAACGGAGAACCAC

Table S3. Primary and seconary antibodies used for immunocytochemical analyses.

Primary Antibody	Source	Product Code	Dilution
Goat anti-SOX17	R&D Systems, Inc.	AF1924	1:100
Mouse anti-OCT3/4	Santa Cruz Biotechnology, Inc.	sc-5279	1:100
Goat anti-PDX1	R&D Systems, Inc.	AF2419	1:100
Goat anti-SOX9	EMD Milipore Cop., USA	AB5535	1:100
Rabbit anti-MAFA	Abcam	Ab26405	1:100
Mouse anti-NKX6.1	Developmental Studies Hybridoma Bank	F55A1-c	1:100
Guinea Pig anti-INSULIN	Dako Cytomation Japan	A0562	1:10
Mouse anti-GLUCAGON	Sigma-Aldrich	G2654	1:100
**Secondary Antibody**	**Source**	**Product Code**	**Dilution**
Alexa 568 donkey anti-goat IgG	Invitrogen	A11057	1:1000
Alexa 647 donkey anti-goat IgG	Jackson ImmunoResearch Inc.	124186	1:1000
Alexa 488 donkey anti-goat IgG	Jackson ImmunoResearch Inc.	124477	1:1000
Alexa 488 donkey anti-mouse IgG	Jackson ImmunoResearch Inc.	715-546-150	1:1000
Alexa 568 donkey anti-mouse IgG	BIOTIUM	CF568	1:1000
Alexa 488 donkey anti-guinea pig IgG	Jackson ImmunoResearch Inc.	115-605-164	1:1000
Alexa 647 donkey anti-mouse IgG	Jackson ImmunoResearch Inc.	706-546-148	1:1000

Biology Open • Supplementary information
